# The Overexpression of Tβ4 in the Hair Follicle Tissue of Alpas Cashmere Goats Increases Cashmere Yield and Promotes Hair Follicle Development

**DOI:** 10.3390/ani10010075

**Published:** 2019-12-31

**Authors:** Bai Dai, Hao Liang, Dong-dong Guo, Zhao-wei Bi, Jian-long Yuan, Yong Jin, Lei Huan, Xu-dong Guo, Ming Cang, Dong-jun Liu

**Affiliations:** State Key Laboratory of Reproductive Regulation and Breeding of Grassland Livestock, School of Life Sciences, Inner Mongolia University, Hohhot 010070, China; daibai007@sina.com (B.D.); lianghao@imu.edu.cn (H.L.); 18847763789@sina.cn (D.-d.G.); lianmengnianhua@126.com (Z.-w.B.); 15904883238@163.com (J.-l.Y.); jin_519@163.com (Y.J.); huanlei320@163.com (L.H.); xudguo@163.com (X.-d.G.); cangming@imu.edu.cn (M.C.)

**Keywords:** Tβ4, secondary hair follicle, cashmere goat, SCNT

## Abstract

**Simple Summary:**

Cashmere goats have double coats consisting of non-modulated fine inner hairs or cashmere fibers produced by secondary hair follicles (SHFs) and guard hairs produced by primary hair follicles (PHFs). Cashmere is an important economic product worldwide. The world market for cashmere is increasing while the current production of cashmere is limited. Thymosin beta-4 (Tβ4), a 4.9 kDa protein, contains 43 amino acids. Here, we investigated whether Tβ4 overexpression would increase SHF numbers, and thus improve the cashmere yield. We produced *Tβ4* transgenic goats using a tissue-specific overexpression strategy. The Tβ4 overexpression (Tβ4-OE) goats had increased hair follicle development and higher cashmere yields than the wild type (WT) goats. The development of this goat model is not only valuable as a framework for future studies of the mechanism of goat follicular development, but will also lead to the improvement of economically important cashmere traits.

**Abstract:**

Increased cashmere yield and improved quality are some goals of cashmere goat breeding. Thymosin beta-4 (Tβ4) plays a key role in the growth and development of hair follicles. For the past ten years, we have evaluated the role of Tβ4 by establishing a flock of 15 cashmere goats that specifically overexpress the *Tβ4* gene in the hair follicles. These Tβ4 overexpression (Tβ4-OE) cashmere goats had more secondary hair follicles than the WT goats and produced more cashmere. Meanwhile, combined analysis of the skin transcriptome and proteome in cashmere goats suggested that Tβ4 may affect hair growth by interacting with keratin type II cytoskeletal 4 epidermal (KRT4) to mediate the extracellular signal-regulated protein kinase (ERK) signaling pathway, thereby promoting the development of secondary hair follicles, and consequently, increasing cashmere yield. Thus, the specific overexpression of Tβ4 in the hair follicles of cashmere goats effectively increased the cashmere yield.

## 1. Introduction

The cashmere goat is one of the most important genetic resources in China [1,2,3]. Alpas cashmere goats live on the Inner Mongolian Plateau, which is characterized by its fragile ecological conditions, intense temperature changes, sparse vegetation, sandy soils, and high winds [4]. After decades of artificial selection, cashmere goats have become the primary goat breed used for the production of cashmere [5,6]. Cashmere, which has become internationally known as soft gold, is an important textile with high economic value [7,8]. Thus, much effort has been directed toward the development of a new cashmere goat breed that can produce higher yields of finer cashmere [9,10,11,12].

Cashmere is derived from secondary hair follicles (SHFs), which are morphologically distinct from primary hair follicles (PHFs) [13]. In contrast to the PHFs (which produce guard hairs), SHFs do not contain medulla, thereby generating fibers that are soft and delicate [14]. Cashmere goat hair follicles (HFs) exhibit an annual periodicity, having three distinct physical phases per year: anagen (growth; August to October), catagen (regression; after December), and telogen (rest; after February). Cashmere production depends primarily on the area and length of the cashmere fibers. These parameters are determined by the number of SHFs and the point in the hair growth cycle at which the fibers are collected [15,16,17,18]. Indeed, one of the key goals of cashmere goat breeding is to increase the number of SHFs carried by the goat.

Thymosin beta-4 (Tβ4), a 4.9 kDa, pleiotropic, actin-sequestering polypeptide containing 43 amino acids, is associated with various functions, such as cell movement, angiogenesis, wound healing, inflammation, and anti-apoptosis [19,20,21]. As a primary G-actin-sequestering peptide, Tβ4 plays a key role in the protection, regeneration, and remodeling of injured or damaged tissues. In rats and mice, Tβ4 promotes the migration of stem cells downward from the bulge region and its immediate progeny, thereby promoting hair growth [22]. Previously, when investigating the effects of endogenous Tβ4 on hair growth in mice, we found that Tβ4 appeared to regulate proliferation-related signaling by influencing vascular endothelial growth factor (VEGF) expression, thereby increasing the speed of hair growth [23,24]. It is, therefore, possible that Tβ4 overexpression might increase the HF number in cashmere goats, subsequently increasing cashmere production.

In addition to traditional breeding methods, more efficient transgenic technologies have recently become widely used to generate transgenic animals. Somatic cell nuclear transfer (SCNT) can be used to produce a large number of embryos from genetically elite animals [25,26]. Reproductive cloning by SCNT using genetically-modified donor cells is a valuable method to propagate animals with desirable traits [9,10,27,28]. Meanwhile, SCNT technology holds great potential for stem cell biology and human therapeutics [29]. This mature technology has been successfully applied to 23 mammalian species [30], including sheep, mice, cattle, pigs, cats, rats, and dogs.

In this study, we aimed to use SCNT to generate HF tissue-specific activated Tβ4 overexpression (Tβ4-OE) goats and to explore whether the Tβ4 could be used to regulate hair follicle growth and development in cashmere goats. Our overarching goal was to develop a framework for the generation of genetically uniform Tβ4-OE goats, which could then be used as the basis for a program dedicated to breeding cashmere goats that produce high yields of high-quality cashmere.

## 2. Materials and Methods 

### 2.1. Animals

All experiments followed the National Research Council Guide for the Care and Use of Laboratory Animals (SYXK 2014-0002). All protocols were approved by the Institutional Animal Care and Use Committee of Inner Mongolia University. All goats were kept at the Inner Mongolia YiWei White Cashmere Goat Limited Liability Company.

### 2.2. Design and Construction of the Vectors

The goat Tβ4 coding sequence was cloned from goat cDNA (NM_001002885). To construct the target vector, the goat Tβ4 coding sequence was ligated with the KAP6.1 promoter. The KAP6.1-Tβ4 fragment was then cloned into the pCDsRed2-1 vector using T4 DNA ligase (TaKaRa Bio, Shiga, Japan). The resulting Tβ4-OE vector was digested and linearized with SacI (Thermo Fisher Scientific, Munich, Germany).

### 2.3. Cell Culture, Transfection, and Selection

To improve the cloning efficiency, we chose goat fibroblasts (GFbs) isolated from both 35-day-old male and female fetal Alpas cashmere goats as donor cells [31]. We then transfected ~5 × 105 GFbs with 3 μg of the Tβ4-OE vector. The GFbs were subsequently screened with 800 μg/mL G418 (Hyclone, Munich, Germany) for 12 days. Individual colonies were identified by sequencing the PCR products covering the target loci.

### 2.4. Karyotype Analysis

We selected one of Tβ4-OE cell lines and one of WT cell lines for the karyotype analysis. We began with a short-term culture of cells derived from each specimen. After a period of cell growth and multiplication, the dividing cells were arrested during metaphase by adding of 0.1 µg/mL colchicine and incubating for 4 h at 37 °C in 5% CO_2_ with saturated humidity. Arrested cells were treated with 0.5 mL KCl. The nuclei were then treated with 5 mL of a chemical fixative for 20 min, dropped on a glass slide, and treated with a Giemsa stain for 30 min.

### 2.5. Cell Growth Curves

We measured the growth of the resulting polyclonal cells by seeding the cells in 24-well plates at a density of 1 × 10^4^ cells/well. Cells were counted every 24 h using a hemocytometer (Qiujing Co., Shanghai, China) for 8 days. Three wells were counted and averaged at each time point; these means were then plotted to calculate the cell growth curve [32].

### 2.6. Generation of Cloned Goats via SCNT

SCNT was performed as described previously [31]. Briefly, oocytes used for SCNT were isolated from goat ovaries collected at a local slaughterhouse (Inner Mongolia YiWei White Cashmere Goat Limited Liability Company Breeding Farm at Ulan Town of Erdos in Inner Mongolia Autonomous Region, China) and cultured by in vitro maturation for 18 to 20 h. The mature oocytes were enucleated, and select polyclonal and WT donor cells were injected into the perivitelline space. The oocyte–donor cell pairs were fused by electric shock. The couplets were fused with two DC pulses of 190 V/mm for 20 μs each, 100 ms apart. The fused embryos were activated in a SOFaa activation solution containing 2 μM IA23187, and cultured in SOFaa medium containing 2 mM 6-DMAP for 4 h [33]. Finally, these embryos were cultured in an embryonic development medium at 38.5 °C for 48 to 50 h. When the embryos had reached the two-to-eight-cell stage, they were transplanted into the oviduct of a recipient and 2–5 embryos were transferred per recipient. We examined the surrogates using ultrasound (Kaixin Co., Xuzhou, China) 90–100 days after embryo transfer to identify the successful pregnancies. All cloned transgenic kids were delivered by natural birth after approximately 150 days of pregnancy. Images of red fluorescent protein expression in the goats were obtained in the dark using portable ultraviolet (UV) light and a digital camera (EOS 600D; Canon, Japan).

### 2.7. PCR Analysis

We genotyped the cells with PCR, using primers designed to identify the integration of the exogenous gene into Tβ4-OE polyclonal cells (Table S1). All PCR conditions are given in Table S1. PCR amplicons were analyzed using 1% agarose gel electrophoresis and purified using the GeneJET Gel Extraction Kit (Thermo Fisher Scientific, Munich, Germany). Purified amplicons were subcloned into pMD19-T vectors (TaKaRa Bio, Shiga, Japan), following the manufacturer’s instructions. Positive clones were selected and sequenced.

### 2.8. Southern Blot Analysis

We assessed the *Tβ4* gene expression in GFbs (isolated from two Tβ4+ goats, two Tβ4- goats, and a WT goat) using a Detection Starter Kit II (Roche, Mannheim, Germany), with the WT goat as the blank control, following the manufacturer’s instructions. In brief, we synthesized a DIG probe by incorporating Digoxigenin-11-dUTP using PCR. The forward and the reverse primers were 5′ATTGAAGAAAACGGAAACGC3′ and 5′GGAACTGGGGGGACAGGATG3′, respectively. The PCR conditions were as follows: 95 °C for 5 min; 35 cycles of 95 °C for 48 s, 60 °C for 30 s, and 72 °C for 40 s, and a final extension at 72 °C for 8 min. We then digested 20 µg of goat genomic DNA overnight with Xbal I. After digestion, the DNA was hybridized with the DIG probe and measured using a chemiluminescent system.

### 2.9. DNA Extraction and qRT-PCR

The genomic goat DNA samples used for the qRT-PCR analysis were extracted and purified using a DNA extraction kit (Promega, Madison, USA). qPCR was performed using SYBR Premix Ex Taq II (TaKaRa Bio, Shiga, Japan) on a 7500 Real-Time PCR System (Applied Biosystems, Munich, Germany) with the following cycling protocol: 95 °C for 30 s; 40 cycles of 95 °C for 5 s and 60 °C for 31 s; 95 °C for 15 s; 60 °C for 1 min; and 95 °C for 15 s. qPCR results were analyzed with qTOWER2-0. Glucagon was used as the reference gene.

### 2.10. RNA Extraction and qRT-PCR

Total RNA was isolated from goat skin tissue samples with RNAiso Plus* (TaKaRa Bio, Shiga, Japan). cDNA was synthesized from 1 μg of the total RNA with a PrimeScript RT reagent Kit with gDNA Eraser (Perfect Real Time) (TaKaRa Bio, Shiga, Japan) in a 20 μL reaction volume, following the manufacturer’s instructions. qPCR was performed using SYBR Premix Ex Taq II (TaKaRa Bio, Shiga, Japan) on a 7500 Real-Time PCR System (Applied Biosystems, Munich, Germany) with the following cycling protocol: 95 °C for 30 s; 40 cycles of 95 °C for 5 s, 60 °C for 34 s, and 95 °C for 15 s; 60°C for 1 min; 95 °C for 30 s; and 60 °C for 15 s. The relative gene expression was calculated using the 2^−ΔΔCt^ method, with GAPDH as the reference gene (see Table S2 for the primers used).

### 2.11. Western Blot Analysis

Skin tissue samples were obtained as described above and prepared for SDS-PAGE. The total proteins were separated using SDS-PAGE and then electroblotted onto a polyvinylidene difluoride membrane. After blocking in 5% non-fat milk for 1 h at room temperature, the membranes were incubated overnight at 4 °C with primary antibodies (1:1000 dilution; Abcam, Cambridge, UK). After incubation, the membrane was rinsed sequentially with phosphate-buffered saline and phosphate-buffered saline containing 0.05% Tween-20 solution. Subsequently, the membrane was treated with secondary antibody. (1:1000 dilution; Abcam, Cambridge, UK). The protein bands were visualized with a Pierce ECL western blotting substrate (Thermo Fisher Scientific, Munich, Germany), using the Tanon 5200 (Tanon, Shanghai, China) detection system. GAPDH was used as the loading control [31,34].

### 2.12. Hematoxylin-Eosin (H&E) Staining

Skin tissues obtained from cloned goats and wild-type goats of the same age were prefixed with 4% paraformaldehyde for 48 h, dehydrated in a series of alcohol concentrations, transferred into xylene, and embedded in paraffin. We cut 5 mm sections from each embedded tissue sample, and stained the sections with H&E. Stained sections were dehydrated and sealed with a cover slip.

### 2.13. RNA Sequencing Alignment and Transcriptomic Analysis

Skin tissue samples were obtained from the backs and abdomens of Tβ4-OE and WT goats and stored in liquid nitrogen. Each sample was ground in liquid nitrogen, and the total RNA was extracted using RNAiso Plus* (TaKaRa Bio, Shiga, Japan), following the manufacturer’s instructions. RNA integrity and purity were assessed using an Agilent 2100 Bioanalyzer (Agilent Technologies, Santa Clara, CA, USA).

We generated sequencing reads of 12 mRNA libraries on a HiSeq ×10 platform (Illumina, San Diego, CA, USA). After removing reads with >5% uncertain bases, >5 bp of adaptor sequences, or >15% low quality bases (Q-score ≤ 19), we retained >6 Gb of clean data for each sample. The reads were aligned using Bowtie2 v2.2.3 [35] based on the Capra_hircus_ARS1 reference genome from the NCBI database (ftp://ftp.ncbi.nlm.nih.gov/genomes/all/GCF/001/704/415/GCF_001704415.1_ARS1/GCF_001704415.1_ARS1_genomic.fna.gz). We successfully aligned 88.89–98.13% of the paired reads with the concordant alignments to the reference genome.

The expected fragments per kilobase of transcript sequence, per million base pairs sequenced (FPKM) for each gene was calculated to estimate the expression, and DESeq2 v1.6.3 [36] was used to identify the differentially expressed genes (DEGs); the genes were considered differentially expressed when q ≤ 0.05 and |log2_ratio| ≥ 1.

### 2.14. Co-Immunoprecipitation (Co-IP)

Co-immunoprecipitation was performed according to the instructions of the Millipore Catch and Release kit. Briefly, samples containing 1–3 mg total protein were precleared with Protein A/G Sepharose for 30 min. The cleared lysates were incubated with 4 mg anti-Tβ4 at 4 °C for at least 2 h, and up to overnight [37].

### 2.15. Protein Mass Spectrometry (MS) Analysis

The Co-IP product of Tβ4 was used for SDS-PAGE. Normal IgG was used as the control. Following Coomassie brilliant blue staining, the SDS-PAGE bands were compared, and the bands that exhibited changes following MS (performed by BGI, Beijing, China) were excised [38].

### 2.16. Cell Proliferation Assay

Cells were incubated with an EdU reagent (Ribobio, Guangzhou, China) at 48 h post-transfection, and a permeabilization buffer was added 24 h later. The cells were then washed with PBS. After staining with Apollo solution (Ribobio, Guangzhou, China) for 30 min, the cells were observed using fluorescence microscopy.

### 2.17. Animal Management and Measurements

According to the feeding standards of large experimental mammals, we managed the cashmere goats and regularly monitored their quality. The body weight of each Tβ4-OE goat was determined at birth, with WT goats of the same age serving as controls. Body weight, cashmere clean weight, cashmere thickness, and fiber length, were monitored yearly until the goat died.

## 3. Results

### 3.1. Generation of Tβ4-OE Alpas Cashmere Goats

**Figure 1:**
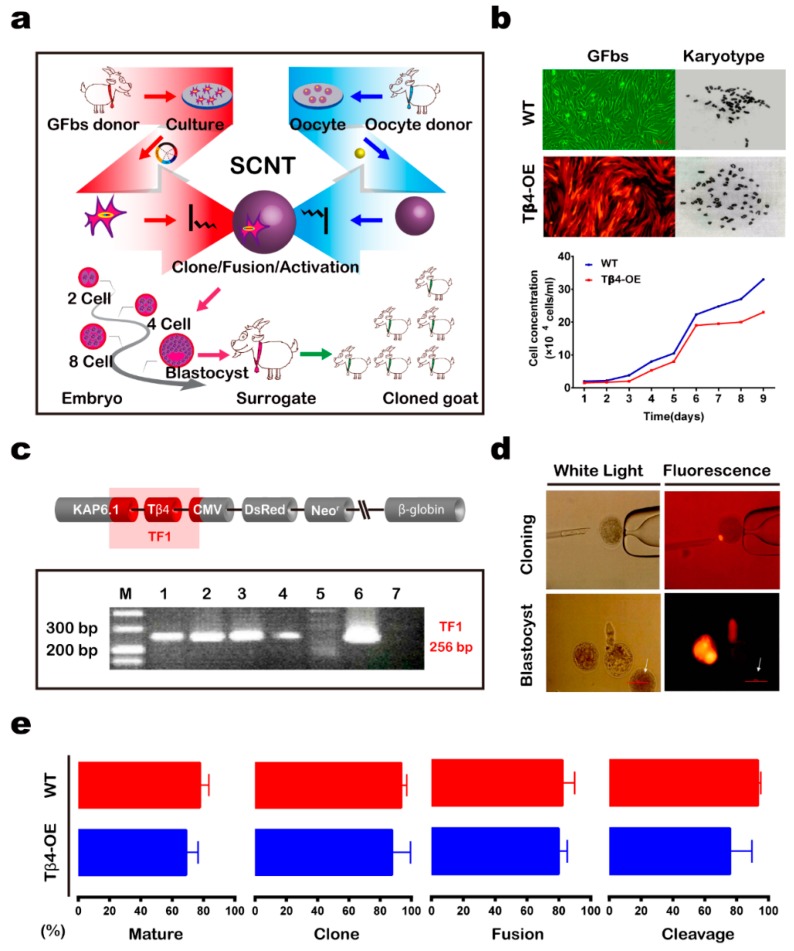
Generation of Tβ4-overexpression (Tβ4-OE) Alpas cashmere goats. (**a**) Schematic of the procedure used to produce Tβ4-OE goats via somatic cell nuclear transfer (SCNT). (**b**) Morphology, karyotype, and growth of WT and Tβ4-OE goat fibroblasts (GFbs). (**c**) Genotype identification of WT and Tβ4-OE GFbs. Lane M, DL500 DNA marker; Lanes 1–4, PCR amplicons of the *Tβ4* gene from the four transgenic GFbs; Lane 5, PCR amplicons of the *Tβ4* gene from the GFbs; Lane 6, PCR amplicons of the *Tβ4* gene from the pCDsR-KT expression vector; Lane 7, PCR amplicons of the *Tβ4* gene from the blank control. (**d**) The Tβ4-OE cloned embryos at various developmental stages. White arrow: blastocyst. Scale bar: 100 μm. (**e**) Comparison between Tβ4-OE (red) and wild type (WT) (blue) cloned embryos at various developmental stages.

We used transgenic cells for producing cashmere goats that specifically overexpressed the *Tβ4* gene in their hair follicles (Figure 1a). First, we constructed a Tβ4 expression vector (pCDsR-KT) carrying the goat cDNA for both Tβ4 (driven by the hair follicle-specific KAP6.1 promoter, which targets both the primary and secondary hair follicles) and the red florescent gene DsRed2 (driven by the cytomegalovirus promoter). This expression vector was transfected into goat fetal fibroblasts (GFbs) derived from 35-day-old male and female fetuses via lipofection. After incubation with G418, 249 (M: 63; F: 186) cell clones remained. We then expanded groups of 15–20 cell clones to form 12 polyclonal cells, which exhibited normal karyotypes and growth (Figure 1b). After propagation and genotyping of the Tβ4-OE polyclonal cells, we found that four polyclonal cell genomes (33.33%) had integrated the pCDsR-KT vector (Figure 1c). 

**Figure 2:**
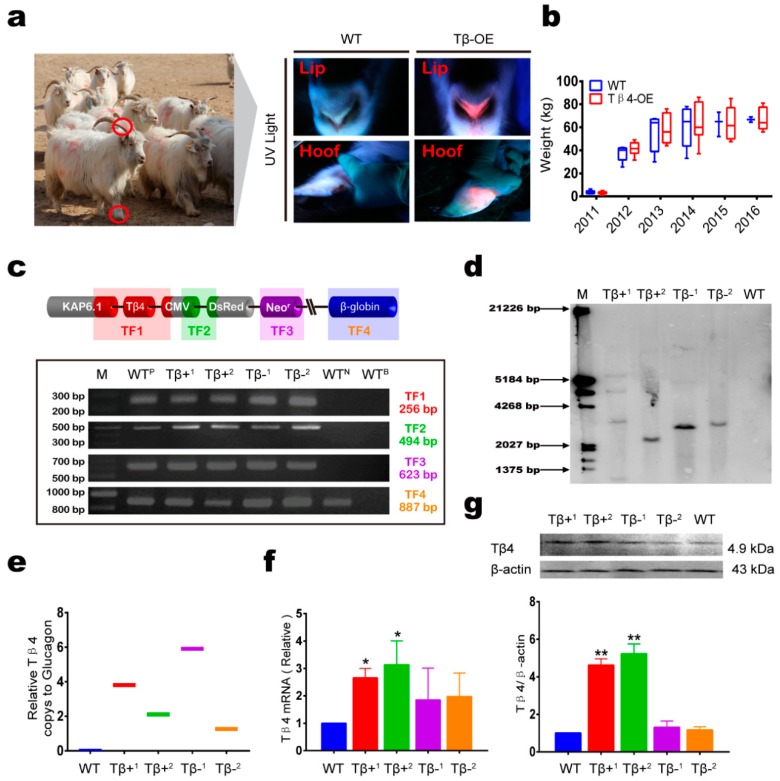
Identification of Tβ4 overexpression (Tβ4-OE) Alpas Cashmere goats. (**a**) Tβ4-OE cashmere goat, showing characteristic red lips and hooves. (**b**) Body weight of wild type (WT, *n* = 8) and Tβ4-OE (*n* = 23) goats from at birth to six years old. Blue boxes are controls; red boxes are Tβ4-OE goats produced via SCNT. (**c**) PCR analysis showing *Tβ4* gene insertion. WT^P^, positive control; WT^N^, negative control; WT^B^, blank control. (**d**) Southern blot and (**e**) absolute quantitative PCR showing Tβ4 copies numbers. (**f**) qPCR and (**g**) western blot showing Tβ4 expression in WT and Tβ4-OE hair follicles to assess the expression of Tβ4. Asterisks indicate that mean expression was significantly different from the control (* *p* < 0.05; ** *p* < 0.01). Bars indicate the means of the three replicates; error bars indicate the standard errors of the means.

Second, four Tβ4-OE polyclonal cells and three wild-type (WT) cell clones were chosen as donors for the SCNT (Figure 1d). We compared embryos cloned from the Tβ4-OE polyclonal cells and the WT cells at various developmental stages (Figure 1e) (Table S3). Cloned embryos from the four Tβ4-OE polyclonal cells were transferred into 241 surrogates. Ultrasonography, 90–100 days after embryo transfer, indicated that 19 surrogates had become pregnant. Four of these surrogate embryos developed to term. In 2010, the 15 successful surrogate mothers gave birth to fifteen Tβ4-OE kids (seven males and eight females; Tables S4–S7). All kids presented with the expected red lips and hooves (Figure 2a). Cloned WT embryos were then transferred into 56 surrogates. Ultrasonography, 90–100 days after the embryo transfer, indicated that eight surrogates had become pregnant, and three of the surrogates’ embryos developed to term. In 2010, five surrogate mothers gave birth to five WT kids (three males and two females; Table 1; Tables S8–S11). 

Third, we monitored the body weights of all cloned cashmere goats for 6 years (Figure 2b); these body weights were not significantly different between the Tβ4-OE and WT goats (*p* > 0.05).

### 3.2. Ectopic Expression of Tβ4 in Tβ4-OE Alpas Cashmere Goats

Tβ4 is expressed in the hair follicles throughout the adult life of the goat. We next investigated whether Tβ4 was overexpressed in the hair follicles of the Tβ4-OE goats by comparing the *Tβ4* gene expression and genotypes among two Tβ4-OE cashmere goats with significantly improved cashmere yield (Tβ4+), two Tβ4-OE cashmere goats with no significant increase in cashmere yield (Tβ4-), and a wild type cashmere goat (WT). Genotyping indicated that all Tβ4-OE goat genomes had integrated the pCDsR-KT vector (Figure 2c). Southern blotting and absolute quantitative PCR showed that all Tβ4-OE goats carried more *Tβ4* copy numbers than the WT goat (Figure 2d,e). However, our quantitative PCR (qPCR) and western blot analyses indicated that the Tβ4 protein and mRNA expression levels were higher in the Tβ4+ goats compared to the WT goats, but these levels for the Tβ4^–^ goats were not significantly higher than those of the WT goats (Figure 2f,g). In addition, we also performed immunofluorescence testing on the samples. In Tβ4+, there was more Tβ4 expression in the hair follicles than in WT. In the WT and Tβ4^–^ goats, there was little difference in Tβ4 expression in the hair follicles (HFs) (Figure S1). We compared Tβ4-labeled fluorescent cell numbers between the WT cell lines and the GFbs lines that we isolated and established. We identified 2.1-fold more Tβ4-labeled fluorescent cells in the Tβ4-OE cell lines compared to the WT cell lines (a 2.1-fold increase; Figure S2).

### 3.3. Cashmere Yield and SHF/PHF Ratio Increased in Tβ4-OE Alpas Cashmere Goats

**Figure 3:**
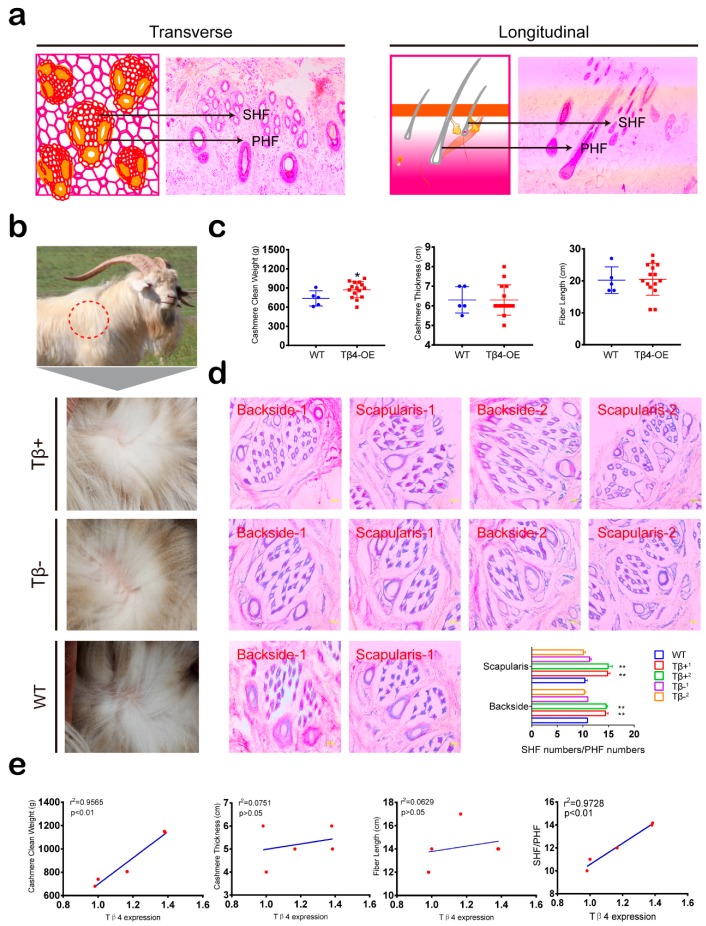
Data showing that the overexpression of Tβ4 increased secondary hair follicle (SHF) number and cashmere yield. (**a**) H&E staining of hair follicle in transverse and longitudinal section. Arrows indicate the primary hair follicle (PHF) and the SHF. (**b**) Comparison of cashmere growth in different goats. (**c**) Cashmere clean weight, cashmere thickness, and fiber length in 15 Tβ4 overexpression (Tβ4-OE) and five wild type (WT) goats at two years old (* *p* < 0.05). (**d**) H&E staining of backside and scapularis skin from Tβ4-OE and WT goats. Scale bar: 100 μm (** *p* < 0.01). (**e**) The correlation between Tβ4 expression and cashmere clean weight, cashmere thickness, fiber length, and the cashmere to hair ratio (SHF/PHF).

Unlike other breeds of cashmere goats, the hair follicles of Alpas cashmere goats are typically composed of three primary hair follicles and several secondary hair follicles (Figure 3a). Cashmere clean weight, thickness, and fiber length collectively indicate cashmere yield. We thus investigated the effects of ectopic Tβ4 expression on hair follicle tissues by measuring these indicators. Although yearly cashmere yields differed among individual goats, the average cashmere yield per goat across the 15 Tβ4-OE goats was generally higher than that of the four WT goats (an ≈100–150 g increase). Specifically, the average yearly cashmere yield of the Tβ+ goats was significantly greater than that of the WT goats (*p* < 0.01), while the average yearly cashmere yield per Tβ4- goat was not significantly different from that of the WT goats (*p* > 0.05). No significant differences in cashmere thickness or fiber length were found between any Tβ4-OE and WT goats (Figure 3b,c). To investigate the difference in cashmere yield among the Tβ4+, Tβ4-, and WT goats, we analyzed the hair follicles of goats from the three groups. Hematoxylin-eosin (H&E) staining of the back and scapula skin tissues of all goats showed that the average SHF/PHF ratio of the Tβ4+ goats was significantly greater than the average SHF/PHF ratios in the WT and Tβ4- goats (Figure 3d). Statistical analysis showed that Tβ4 expression levels in the hair follicle tissues were significantly correlated with cashmere clean weight (*p* < 0.01) and SHF/PHF ratio (*p* < 0.01) but were not significantly correlated with cashmere thickness or fiber length (*p* > 0.05 Figure 3e).

### 3.4. Tβ4 May Affect Hair Growth by Interacting with KRT4 to Mediate ERK Signaling Pathway

**Figure 4:**
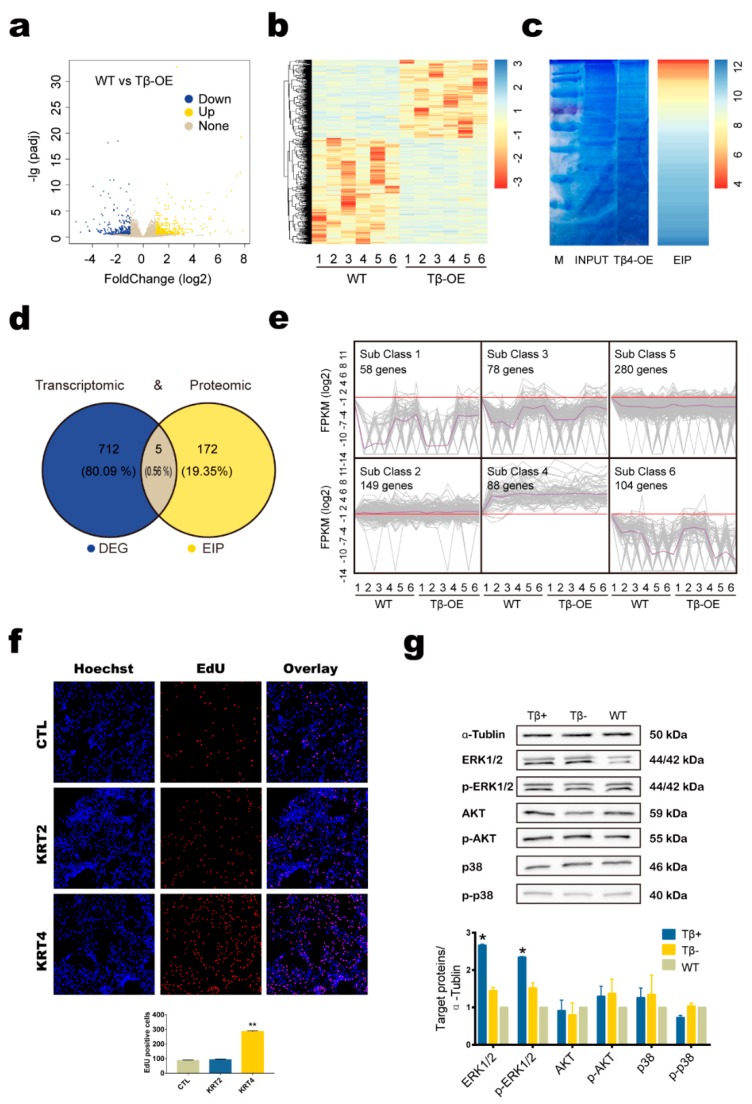
The integrated transcriptomic and proteomic strategy used to investigate the mechanisms underlying the increase in cashmere yield and hair follicle development associated with of Tβ4. (**a**) The volcano plot of all genes in the skin samples from the Tβ4 overexpression (Tβ4-OE) cashmere goats and WT cashmere goats, showing genes with a >2-fold difference and an adjusted *p* < 0.01 among the groups. (**b**) The heat map of the gene expression profiles in the skin samples from the Tβ4-OE and wild type (WT) cashmere goats. The colored bars illustrate relative expression. In the map, each sample group is clustered. (**c**) HeLa cells transiently overexpressing Tβ4 were immunoprecipitated with the anti-Tβ4 antibody for Coomassie Brilliant Blue staining; possible proteins that interact with Tβ4 were identified using mass spectrometry. (**d**) Venn diagram illustrating the overlap of five genes between the transcriptome-identified DEGs and the proteome-identified enriched interacting proteins (EIP). (**e**) A cluster analysis of DEGs identified among the skin samples of the Tβ4-OE and WT goats and functional categorization of the recovered subclusters. (**f**) Results of the EdU staining assay, showing cell proliferation in secondary hair follicle dermal papilla cells (SHF-DPCs) overexpressing keratin type II cytoskeletal 2 epidermal (KRT2) or keratin type II cytoskeletal 4 (KRT4). Hoechst reagent stains all cell nuclei, while the EdU (5-Ethynyl-2′-deoxyuridine) reagent only stains the newly proliferated cell nuclei (** *p* < 0.01). (**g**) Detection of the proliferation-related signaling pathways in Tβ4-OE cashmere goats with significantly improved cashmere yield (Tβ4+), Tβ4-OE cashmere goats with no significant increase in cashmere yield (Tβ4-), and wild type cashmere goats (WT) (* *p* < 0.05).

To identify DEGs in the skin samples of the Tβ4-OE and WT cashmere goats, we compared the transcriptomes of the three groups and identified 717 DEGs (Figure 4a,b). We next identified the proteins affected by *Tβ4* gene expression using co-immunoprecipitation (Co-IP) in combination with mass spectrometry (MS). The proteins were separated with SDS-PAGE electrophoresis, and the protein bands were visualized using Coomassie blue staining. Using MS, we identified 177 proteins that may interact with Tβ4 (Figure 4c). Five candidate genes were identified by both the transcriptomic and the proteomic analyses: ALBINO3-like protein 1 (ALB), COP9 signalosome complex subunit 3 (CSN3), protein S100-A8 (S100A8), keratin type II cytoskeletal 2 epidermal (KRT2), and keratin type II cytoskeletal 4 (KRT4) (Figure 4d). A KEGG pathway analysis revealed that the DEG were mainly enriched in pathways in arachidonic acid metabolism, linoleic acid metabolism, and nicotine addiction. Meanwhile, the KEGG pathway analysis revealed that the enriched interacting proteins (EIP) were mainly enriched in ribosomes, the biosynthesis of amino acids, and carbon metabolism (Table 2).

A k-means cluster (k-cluster) analysis was then performed to characterize the expression profiles of the 717 DEGs identified in the skin samples. The K-cluster analysis classified all DEGs identified across the Tβ4-OE and WT skin samples into six subclusters (Figure 4e). As genes with similar expression patterns are most likely regulated similarly, we compared the subclusters into which each candidate gene fell: ALB was classified in subcluster 3, CSN3 was classified in subcluster 6, S100A8 was classified in subcluster 2, and KRT2 and KRT4 were classified in subcluster 4. KRT2 and KRT4 are KRT homologs, which are well-known fibrous structural proteins found in hair, nails, horns, hooves, wool, feathers, and the outermost epithelial cells. As these proteins may be related to hair follicles, they were examined in further detail. This analysis indicated that the genes in subcluster 4 were primarily associated with cell proliferation, cell growth, and cell differentiation.

The EdU (5-Ethynyl-2′-deoxyuridine) assays showed that cell proliferation increased remarkably after KRT4 was overexpressed in secondary hair follicle dermal papilla cells (SHF-DPCs). However, cell proliferation in the SHF-DPCs was unaffected by KRT2 overexpression (Figure 4f). We also investigated the proliferation-related signaling pathways (i.e., AKT, ERK, and p38) using skin samples from Tβ4+, Tβ4-, and WT goats. In the Tβ4+ goats, ERK protein expression and phosphorylation levels were significantly higher than those in the WT goat (*p* < 0.05). However, the ERK protein expression and phosphorylation levels in the Tβ4- goats were not significantly different from those in the WT goat (Figure 4g).

## 4. Discussion

The improvement of cashmere yield and quality are two of the goals of cashmere goat breeding. Here, we demonstrated that the overexpression of Tβ4, which is involved in many critical biological processes, including cell proliferation, cell differentiation, and angiogenesis [39,40,41], promotes hair follicle development and increased cashmere yield. Tβ4 stimulates new hair growth by inducing the migration of hair-follicle stem cells [42]. We found that Tβ4 overexpression led to a relative increase in the number of SHFs (i.e., a greater SHF/PHF ratio), consistent with previous reports that indicated that Tβ4 overexpression in mouse hair follicles increased hair density [23,24]. Importantly, the Tβ4 transgenic goats were healthy, with normal growth rates, normal activity levels, and no obvious behavioral defects. This suggests that the marker genes such as DsRed and neo we inserted into the genome had no effect on the growth and development of cashmere goats. Thus, despite environmental and experimental challenges, the current method can ensure the safety of transgenic animals. However, the low production efficiency of SCNT is still the bottleneck restricting to its wide application. How to further improve the method should be our focus. In addition, we counted thickness and fiber length to measure cashmere yield. Although they cannot directly affect the cashmere yield, they can indirectly reflect the cashmere yield. These two indexes are also important reference indexes for cashmere quality. Our result suggests that increasing the yield of cashmere did not reduce the quality of cashmere.

In this study, we used the cashmere goat as a model with which to study the effects of Tβ4 on hair follicles in vivo. Five candidate genes were identified by both the transcriptomic and proteomic analyses, and two of these (KRT2 and KRT4) were shown to have similar expression patterns by k-means clustering. As KRT homologs are closely related to hair growth [43,44,45], we separately overexpressed KRT2 and KRT4 in SHF-DPCs and found that KRT4 increased the growth of SHF-DPCs. In addition, our investigation of proliferation-related signaling pathways showed that the ERK signaling pathway was significantly enhanced in Tβ4+ goats, which is consistent with previous reports that this signaling pathway is involved in the regulation of hair follicle development in a variety of model animals [41,46,47]. This suggested that Tβ4 might bind to KRT4 to mediate the ERK signaling pathway, thereby affecting hair follicle development. This result provides a basis for the study of the molecular mechanisms underlying these effects. In addition, it is always the most important task in the field of animal breeding to breed offspring that can stably inherit the fine traits of their parents. In light of having initially produced 15 Tβ4 overexpression (Tβ4-OE) cashmere goats, we have successfully established a flock of 23 offspring (six via SCNT and 17 via natural mating (NM). We are also working on studies of genetic stability across generations [48]. However, the desired trait (cashmere yield) exhibited lower fixation in the line-bred offspring compared to the SCNT offspring. It may be due to environmental factors or the organism itself, but part of the randomly integrated Tβ4 of NM offspring underwent methylation modification, leading to a weakened effect on cashmere yield. Our study systematically describes the DNA methylation characteristics between generations of cashmere goats and provides a basis for improving genetic stability.

Genome editing in cashmere goats has many important potential applications with respect to cashmere production. By establishing a Tβ4-OE goat model, we demonstrated that the ectopic expression of Tβ4 promoted hair follicle development and increased cashmere yield. However, the random genomic integration of foreign genes often results in unstable phenotypes, gene silencing, and unpredictable gene expression patterns [49,50]. Here, in some Tβ4-OE goats, cashmere yield was not improved (Tβ4-), indicating that the Tβ4 did not work. One possible explanation for this phenomenon is that, although we integrated Tβ4 into the genome of the cashmere goat, Tβ4 was not overexpressed in the hair follicles of the cashmere goats, likely because methylation in the promoter region of the randomly integrated Tβ4 led to the silencing of Tβ4 expression [51]. Therefore, it would be more efficient to produce animals carrying exogenous genes integrated at specific genomic loci [52]. Recently developed nuclease-mediated genome editing techniques, such as CRISPR/Cas9, provide precise and efficient methods for the genetic editing of cells, tissues, and whole organisms [53]. It is clear that CRISPR-based genome engineering will accelerate improvements in desirable livestock traits, as this technology allows the precise regulation of a target gene [53,54,55]. At present, we are integrating CRISPR/Cas9 with SCNT to breed cashmere goats carrying the *Tβ4* gene knocked in at specific sites. In this way, we hope not only to precisely regulate the target gene, but also to reduce the effects of gene editing on the genome as a whole. In addition, the use of CRISPR/Cas9 avoids the target gene silencing caused by random integration, which will ensure the stable expression of the target gene and the presence of the target trait in all offspring. In our previous study [10], we successfully obtained a Tβ4 knock-in goat without any screening and fluorescent markers using CRISPR/Cas9 technology. The goat exhibited an increase in cashmere yield by 74.5% without affecting the fineness and quality. Meanwhile, we have specifically inserted the *Tβ4* gene into the goat CCR5 locus which has been suggested as a safe and reliable harbor site for knocking in a foreign gene. Our results have shown that the Tβ4 CCR5-knock-in goats have no developmental or other abnormalities. In addition, the site can stably express *Tβ4* genes without affecting the expression of *Tβ4* genes around the integration site.

## 5. Conclusions

In summary, we produced *Tβ4* transgenic goats using a tissue-specific overexpression strategy. The Tβ4-overexpression transgenic goats had increased hair follicle development and higher cashmere yields than the WT goats. The development of this goat model was not only valuable as a framework for future studies of the mechanism of goat follicular development, but also directly led to the improvement of an economically important cashmere goat trait. However, the beneficial effects of ectopic Tβ4 overexpression on cashmere production efficiency and goat physiology must be verified among larger numbers of goats across several different commercial goat breeds.

## Figures and Tables

**Table 1:** Goat birth statistics.

Group	SCNT Embryos (n)	Embryos Transferred (n)	Surrogates (n)	Pregnancies (n)	The Rate of Pregnancy (%)	The Number of Birth (n)	The Male ( 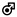 ) Ratio (%)	The Female ( 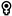 ) Ratio (%)	The Rate of Cloning Efficiency (%)
WT	360	360	56	8	14.2	5	60.0	40.0	1.39
Tβ4-OE	1155	1137	241	19	8.9	15	46.7	53.3	1.32

**Table 2:** Comparison of DEG and EIP pathway enrichment analysis.

Type	Pathway Name	Count	*p*-Value	FDR ^3^
DEG ^1^	Arachidonic acid metabolism	10	1.75846 × 10^−5^	0.002971802
	Linoleic acid metabolism	7	1.17096 × 10^−5^	0.002971802
	Nicotine addiction	6	0.00036797	0.024874782
	Steroid hormone biosynthesis	8	0.000307903	0.024874782
	Taste transduction	7	0.000363475	0.024874782
	Natural killer cell mediated cytotoxicity	12	0.000649358	0.034851257
	Neuroactive ligand-receptor interaction	15	0.000721772	0.034851257
	Retinol metabolism	8	0.001021739	0.043168488
EIP ^2^	Ribosome	11	3.28496 × 10^−8^	3.57362 × 10^−5^
	Biosynthesis of amino acids	7	1.12507 × 10^−5^	0.012238664
	Carbon metabolism	8	1.23556 × 10^−5^	0.013440547
	Glycolysis/Gluconeogenesis	6	8.69038 × 10^−5^	0.094499892
	Biosynthesis of antibiotics	9	0.000108387	0.117847957

^1^ DEG, differentially expressed genes; ^2^ EIP, enriched interacting proteins; ^3^ FDR, false discovery rate.

## Supplementary Materials

The following are available online at https://www.mdpi.com/2076-2615/10/1/75/s1, Figure S1: Representative images of Tβ4 overexpression (Tβ4-OE) cashmere goat hair follicles stained with anti-Tβ4 and DAPI, Figure S2: Immunofluorescent detection of Tβ4 expression using the Tβ4 antibody, Table S1: List of PCR primer sequences, Table S2: List of qPCR primer sequences, Table S3: Early in vitro development of cloned embryos from different cell lines, Table S4: Statistics on the Tβ4 overexpression (Tβ4-OE) Embryo of P0, Table S5: Statistics on the Tβ4 overexpression (Tβ4-OE) Oestrus of P0, Table S6: Statistics on the Tβ4 overexpression (Tβ4-OE) Transfer of P0, Table S7: Statistics on the Tβ4 overexpression (Tβ4-OE) Clonal Goat of P0, Table S8: Statistics on the wild type (WT) Embryo of P0, Table S9: Statistics on the wild type (WT) Oestrus of P0, Table S10: Statistics on the wild type (WT) Transfer of P0, Table S11: Statistics on the wild type (WT) Clonal Goat of P0.
