# Supplementary material for: The Overexpression of Tβ4 in the Hair Follicle Tissue of Alpas Cashmere Goats Increases Cashmere Yield and Promotes Hair Follicle Development
Source: Animals (Basel). 2019 Dec 31;10(1):75. doi: 10.3390/ani10010075 (PMC7022706; doi:10.3390/ani10010075)
Supplement: Supplementary file 1 [file animals-10-00075-s001.pdf]

The Overexpression of Tβ4 in the Hair Follicle Tissue of Alpas Cashmere Goats Increases Cashmere Yield and Promotes Hair Follicle Development

Bai Dai [1,†], Hao Liang [1,†], Dong-dong Guo [1], Zhao-wei Bi [1], Jian-long Yuan [1], Yong Jin [1], Lei Huan [1], Xu-dong Guo [1], Ming Cang [1] and Dong-jun Liu [1,*]

[1] State Key Laboratory of Reproductive Regulation and Breeding of Grassland Livestock, School of Life Sciences, Inner Mongolia University, Hohhot, P.R.China

* Correspondence: nmliudongjun@sina.com; Tel.: +86- 0471-4995071 (D-J.L.)

† These authors contributed equally to this work.

Supplementary Figures

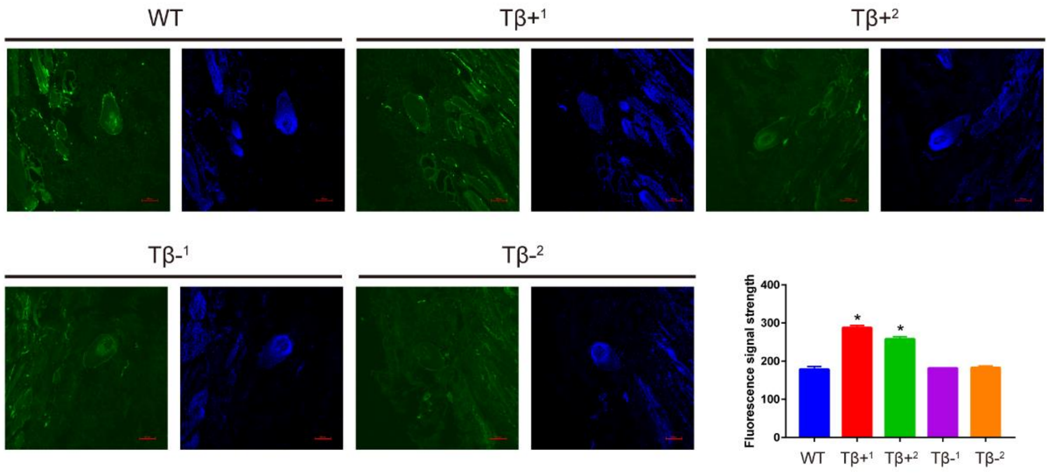

Figure S1 Representative images of Tβ4 overexpression (Tβ4-OE) cashmere goat hair follicles stained with anti-Tβ4 and DAPI. WT: wild type; Tβ4+: Tβ4-OE cashmere goat with significantly improved cashmere yield, Tβ4-: Tβ4-OE cashmere goat with no significant increase in cashmere yield. Scale bar: 30 μm.

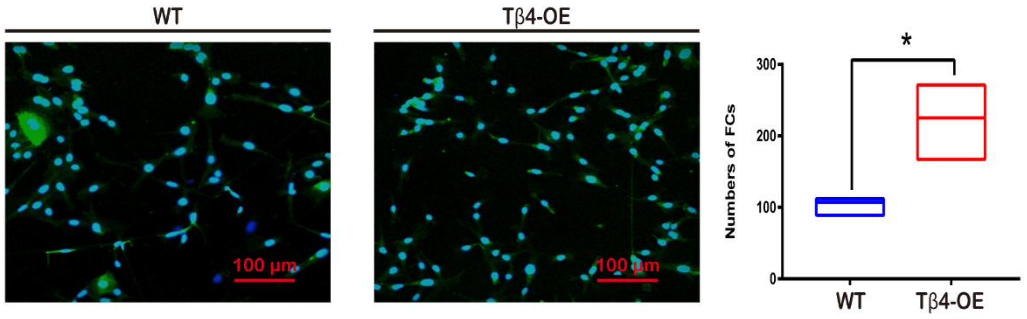

Figure S2 Immunofluorescent detection of Tβ4 expression using the Tβ4 antibody Left: wild type goat; right: Tβ4 overexpression (Tβ4-OE) goat. The histogram further illustrates the fluorescent cell numbers shown in the image (*, $P < 0.05$).

Supplementary Tables

Table S1 List of PCR primer sequences

Site	Primers sequence (5′–3′)	Annealing Temperature (C°)	Time of Elongation (s)	Size of PCR product (bp)
KTC	5'-TCAACAAGCAACACCTCCTA-3'<br>5'-CGGTAATACGGTTATCCACA-3'	55	60	256
CD	5'-TTCCAAAATGTCGTAACAACTCCG-3'<br>5'-TCACCTTGTAGATGAAGCAGCCGTC-3'	60	60	494
NEO	5'-ATGACTGGGCACAACAGACAATCG-3'<br>5'-AGCAATATCACGGGTAGCCAACGC-3'	55	60	623
β-globin	5'-CCTCTAGATTCCCGCTGCTCCTGA-3'<br>5'-AAGAATTCGGGTGACGATGATGGC-3'	65	60	887
Tβ4	5'-CGGGATCCGTATGTCTGACAAACCCGATAT-3'<br>5'-ACGCGTCGACTTACGACTCGCCTGCTTGCT-3'	58	30	166
β-actin	5'-CGGGATCCGTATGGATGATGATATTGCTGC-3'<br>5'-ATAAGAATGCGGCCGCCTAGAAGCATTTGCGGTGGA-3'	60	70	1140

Table S2 List of qPCR primer sequences

Site	Primers sequence (5′–3′)	Annealing Temperature (C°)	Time of Elongation (s)	Size of PCR product (bp)
Tβ4	5'-AAACCCGATATGGCTGAGATTG-3'<br>5'-GCCTGCTTGCTTCTCCTGTT-3'	60	60	117
GAPDH	5’-CCACTTTGTCAAGCTCATTTCCT-3’<br>5’-TCTCTCTTCCTCTCGTGCTCCT-3’	61	60	140

Table S3. Early in vitro development of cloned embryos from different cell lines

Cell lines	Matured oocytes (%)	Nuclear transferred oocytes (%)	Fused cells (%)	Cleavage embryos (%)
Tβ4-OE-A	68 (72.34)	61 (89.71)	45 (73.77)	41 (91.11)
Tβ4-OE-B	70 (81.40)	68 (97.14)	53 (77.94)	49 (92.45)
Tβ4-OE-C	72 (83.72)	69 (95.83)	61 (88.41)	58 (95.08)
Tβ4-OE-D	70 (73.68)	64 (91.43)	57 (89.06)	54 (94.74)
**Overall**	**280 (77.56)**	**262 (93.57)**	**216 (82.44)**	**202 (93.52)**
WT-a	75 (78.13)	71 (94.67)	56 (78.87)	31 (55.36)
WT-b	108 (72.00)	80 (74.07)	70 (87.50)	59 (84.29)
WT-c	106 (64.24)	87 (82.08)	65 (74.71)	54 (83.08)
WT-d	65 (61.32)	65 (100.00)	51 (78.46)	42 (80.77)
**Overall**	**354 (68.47)**	**303 (85.59)**	**242 (79.87)**	**186 (76.54)**

Table S4. Statistics on the Tβ4 overexpression (Tβ4-OE) Embryo of P0

Embryo NO.	Year	Cleavage
100057	2010	48
100058	2010	38
100059	2010	23
100060	2010	28
100061	2010	38
100062	2010	62
100063	2010	84
100064	2010	82
100065	2010	93
100066	2010	53
100067	2010	69
100068	2010	60
100069	2010	60
100071	2010	24
100072	2010	42
100073	2010	42
100078	2010	72
100079	2010	60
100080	2010	28
100081	2010	40
100082	2010	109

Table S5. Statistics on the Tβ4 overexpression (Tβ4-OE)

Oestrus of P0

Oestrus NO.	Surrogate NO.	Date
100408	100323	23-Sep-10
100409	100324	23-Sep-10
100410	100325	23-Sep-10
100411	100326	23-Sep-10
100412	100327	23-Sep-10
100413	100328	23-Sep-10
100414	100329	23-Sep-10
100415	100330	23-Sep-10
100416	100331	23-Sep-10
100417	100332	25-Sep-10
100418	100333	25-Sep-10
100419	100334	25-Sep-10
100420	100335	25-Sep-10
100421	100336	25-Sep-10
100422	100337	25-Sep-10
100423	100338	26-Sep-10
100424	100339	26-Sep-10
100425	100340	26-Sep-10
100426	100341	26-Sep-10
100427	100342	26-Sep-10
100428	100343	27-Sep-10
100429	100344	27-Sep-10
100430	100345	27-Sep-10
100431	100346	27-Sep-10
100432	100347	27-Sep-10
100433	100348	28-Sep-10
100434	100349	28-Sep-10
100435	100350	28-Sep-10
100436	100351	28-Sep-10
100437	100352	28-Sep-10
100438	100353	28-Sep-10
100439	100354	28-Sep-10
100440	100355	28-Sep-10
100441	100356	28-Sep-10
100442	100357	28-Sep-10
100443	100358	28-Sep-10
100444	100359	28-Sep-10
100445	100360	28-Sep-10
100446	100361	29-Sep-10

100447	100362	29-Sep-10
100448	100363	29-Sep-10
100449	100364	29-Sep-10
100450	100365	29-Sep-10
100451	100366	29-Sep-10
100452	100367	29-Sep-10
100453	100368	29-Sep-10
100454	100369	29-Sep-10
100455	100370	29-Sep-10
100456	100371	29-Sep-10
100457	100372	29-Sep-10
100458	100373	29-Sep-10
100459	100374	29-Sep-10
100460	100375	29-Sep-10
100461	100376	29-Sep-10
100462	100377	29-Sep-10
100463	100378	29-Sep-10
100464	100379	29-Sep-10
100465	100380	29-Sep-10
100466	100381	30-Sep-10
100467	100382	30-Sep-10
100468	100383	30-Sep-10
100469	100384	30-Sep-10
100470	100385	30-Sep-10
100471	100386	30-Sep-10
100472	100387	30-Sep-10
100473	100388	30-Sep-10
100474	100389	30-Sep-10
100475	100390	30-Sep-10
100476	100391	30-Sep-10
100477	100392	30-Sep-10
100478	100393	30-Sep-10
100479	100394	30-Sep-10
100480	100395	30-Sep-10
100481	100396	30-Sep-10
100482	100397	30-Sep-10
100483	100398	30-Sep-10
100484	100399	30-Sep-10
100485	100400	30-Sep-10
100486	100401	30-Sep-10
100487	100402	30-Sep-10
100488	100403	30-Sep-10

100489	100404	30-Sep-10
100490	100405	30-Sep-10
100491	100406	30-Sep-10
100492	100407	30-Sep-10
100493	100408	30-Sep-10
100494	100409	01-Oct-10
100495	100410	01-Oct-10
100496	100411	01-Oct-10
100497	100412	01-Oct-10
100498	100413	01-Oct-10
100499	100414	01-Oct-10
100500	100415	01-Oct-10
100501	100416	01-Oct-10
100502	100417	01-Oct-10
100503	100418	01-Oct-10
100504	100419	01-Oct-10
100505	100420	01-Oct-10
100506	100421	01-Oct-10
100507	100422	01-Oct-10
100508	100423	01-Oct-10
100509	100424	01-Oct-10
100510	100425	01-Oct-10
100511	100426	01-Oct-10
100512	100427	01-Oct-10
100513	100428	01-Oct-10
100514	100429	01-Oct-10
100515	100430	01-Oct-10
100516	100431	01-Oct-10
100517	100432	02-Oct-10
100518	100433	02-Oct-10
100519	100434	02-Oct-10
100520	100435	02-Oct-10
100521	100436	02-Oct-10
100522	100437	02-Oct-10
100523	100438	02-Oct-10
100524	100439	02-Oct-10
100525	100440	02-Oct-10
100526	100441	02-Oct-10
100527	100442	02-Oct-10
100528	100443	02-Oct-10
100529	100444	02-Oct-10
100530	100445	02-Oct-10

100531	100446	02-Oct-10
100532	100447	02-Oct-10
100533	100448	03-Oct-10
100534	100449	03-Oct-10
100535	100450	03-Oct-10
100536	100451	03-Oct-10
100537	100452	03-Oct-10
100538	100453	03-Oct-10
100539	100454	03-Oct-10
100540	100455	03-Oct-10
100541	100456	05-Oct-10
100542	100457	05-Oct-10
100543	100458	05-Oct-10
100544	100459	05-Oct-10
100545	100460	05-Oct-10
100546	100461	05-Oct-10
100547	100462	05-Oct-10
100548	100463	05-Oct-10
100549	100464	05-Oct-10
100550	100465	05-Oct-10
100551	100466	05-Oct-10
100552	100467	06-Oct-10
100553	100468	06-Oct-10
100554	100469	06-Oct-10
100555	100470	06-Oct-10
100556	100471	06-Oct-10
100557	100472	06-Oct-10
100558	100473	06-Oct-10
100559	100474	06-Oct-10
100560	100475	06-Oct-10
100561	100476	06-Oct-10
100562	100477	07-Oct-10
100563	100478	07-Oct-10
100564	100479	07-Oct-10
100565	100480	07-Oct-10
100566	100481	07-Oct-10
100567	100482	07-Oct-10
100568	100483	07-Oct-10
100569	100484	07-Oct-10
100570	100485	07-Oct-10
100571	100486	07-Oct-10
100572	100487	07-Oct-10

100573	100488	07-Oct-10
100582	100497	08-Oct-10
100583	100498	08-Oct-10
100584	100499	08-Oct-10
100585	100500	08-Oct-10
100586	100501	09-Oct-10
100587	100502	09-Oct-10
100588	100503	09-Oct-10
100589	100504	09-Oct-10
100590	100505	09-Oct-10
100591	100506	09-Oct-10
100592	100507	09-Oct-10
100593	100508	10-Oct-10
100594	100509	10-Oct-10
100595	100510	10-Oct-10
100596	100511	10-Oct-10
100597	100512	10-Oct-10
100598	100513	10-Oct-10
100599	100514	10-Oct-10
100635	100549	15-Oct-10
100636	100550	15-Oct-10
100637	100551	15-Oct-10
100638	100552	15-Oct-10
100639	100412	15-Oct-10
100640	100553	15-Oct-10
100641	100554	15-Oct-10
100642	100555	15-Oct-10
100643	100556	15-Oct-10
100644	100332	15-Oct-10
100645	100557	15-Oct-10
100646	100333	15-Oct-10
100647	100396	16-Oct-10
100648	100558	16-Oct-10
100649	100341	16-Oct-10
100650	100337	16-Oct-10
100651	100336	16-Oct-10
100652	100350	16-Oct-10
100653	100559	16-Oct-10
100654	100560	16-Oct-10
100655	100561	16-Oct-10
100656	100562	16-Oct-10
100657	100563	17-Oct-10

100658	100564	17-Oct-10
100659	100565	17-Oct-10
100660	100566	17-Oct-10
100661	100385	18-Oct-10
100662	100371	18-Oct-10
100663	100398	18-Oct-10
100664	100567	18-Oct-10
100665	100357	18-Oct-10
100666	100354	18-Oct-10
100667	100367	18-Oct-10
100668	100401	18-Oct-10
100669	100344	18-Oct-10
100670	100359	19-Oct-10
100671	100355	19-Oct-10
100672	100353	19-Oct-10
100673	100568	19-Oct-10
100674	100349	19-Oct-10
100675	100381	19-Oct-10
100676	100376	19-Oct-10
100677	100378	19-Oct-10
100678	100327	19-Oct-10
100679	100363	19-Oct-10
100680	100411	19-Oct-10
100681	100375	19-Oct-10
100682	100362	19-Oct-10
100683	100358	19-Oct-10
100684	100569	19-Oct-10
100685	100570	19-Oct-10
100686	100571	19-Oct-10
100687	100572	19-Oct-10
100688	100573	19-Oct-10
100689	100338	19-Oct-10
100690	100400	19-Oct-10
100691	100352	19-Oct-10

Table S6. Statistics on the Tβ4 overexpression (Tβ4-OE) Transfer of P0

Transfer NO.	Oestrus NO.	Embryo NO.	Date	Embryo of Transfer		Site of Transfer			Number of Transfer		
				n-Cleavage	Cleavage	OPR	OPL	Site	Total	RS	LS
100393	100408	100057	25-Sep-10	10	0	0	2	R	10	0	10
100394	100409	100057	25-Sep-10	0	0	0	0	N	0	0	0
100395	100410	100057	25-Sep-10	5	0	0	1	R	5	0	5
100396	100411	100057	25-Sep-10	7	0	1	1	L	7	7	0
100397	100412	100057	25-Sep-10	8	0	1	0	L	8	8	0
100398	100413	100057	25-Sep-10	0	0	0	0	N	0	0	0
100399	100414	100057	25-Sep-10	8	0	1	0	L	8	8	0
100400	100415	100057	25-Sep-10	10	0	1	2	R	10	0	10
100401	100416	100057	25-Sep-10	0	0	0	0	N	0	0	0
100402	100417	100058	27-Sep-10	7	0	1	0	L	7	7	0
100403	100418	100058	27-Sep-10	7	0	1	0	L	7	7	0
100404	100419	100058	27-Sep-10	0	0	0	0	N	0	0	0
100405	100420	100058	27-Sep-10	6	0	1	1	R	6	0	6
100406	100421	100058	27-Sep-10	6	0	1	1	L	6	6	0
100407	100422	100058	27-Sep-10	6	0	1	1	R	6	0	6
100408	100423	100059	28-Sep-10	4	0	4	0	L	4	4	0
100409	100424	100059	28-Sep-10	5	0	5	0	L	5	5	0
100410	100425	100059	28-Sep-10	5	0	1	0	L	5	5	0
100411	100426	100059	28-Sep-10	5	0	3	2	L	5	5	0
100412	100427	100059	28-Sep-10	4	0	1	0	L	4	4	0
100413	100428	100060	29-Sep-10	6	0	0	1	R	6	0	6

100414	100429	100060	29-Sep-10	6	0	0	2	L	6	6	0
100415	100430	100060	29-Sep-10	6	0	1	1	L	6	6	0
100416	100431	100060	29-Sep-10	5	0	0	1	R	5	0	5
100417	100432	100060	29-Sep-10	5	0	1	0	L	5	5	0
100418	100433	100061	30-Sep-10	2	1	1	0	L	3	3	0
100419	100434	100061	30-Sep-10	2	1	1	1	R	3	0	3
100420	100435	100061	30-Sep-10	2	1	1	1	L	3	3	0
100421	100436	100061	30-Sep-10	2	1	1	1	L	3	3	0
100422	100437	100061	30-Sep-10	2	1	1	0	L	3	3	0
100423	100438	100061	30-Sep-10	2	1	0	1	R	3	0	3
100424	100439	100061	30-Sep-10	2	1	0	0	R	3	0	3
100425	100440	100061	30-Sep-10	3	0	1	1	L	3	3	0
100426	100441	100061	30-Sep-10	3	0	0	2	R	3	0	3
100427	100442	100061	30-Sep-10	3	0	0	2	R	3	0	3
100428	100443	100061	30-Sep-10	2	0	0	1	R	2	0	2
100429	100444	100061	30-Sep-10	3	0	0	1	R	3	0	3
100430	100445	100061	30-Sep-10	3	0	1	0	L	3	3	0
100431	100446	100062	01-Oct-10	3	0	0	1	R	3	0	3
100432	100447	100062	01-Oct-10	3	0	0	1	R	3	0	3
100433	100448	100062	01-Oct-10	3	0	1	1	L	3	3	0
100434	100449	100062	01-Oct-10	3	0	1	0	L	3	3	0
100435	100450	100062	01-Oct-10	3	0	0	1	R	3	0	3
100436	100451	100062	01-Oct-10	3	0	1	1	L	3	3	0
100437	100452	100062	01-Oct-10	3	0	0	1	R	3	0	3
100438	100453	100062	01-Oct-10	3	0	0	2	R	3	0	3

100439	100454	100062	01-Oct-10	3	0	1	1	R	3	0	3
100440	100455	100062	01-Oct-10	3	0	1	0	L	3	3	0
100441	100456	100062	01-Oct-10	3	0	2	0	L	3	3	0
100442	100457	100062	01-Oct-10	3	0	1	0	L	3	3	0
100443	100458	100062	01-Oct-10	3	0	1	0	L	3	3	0
100444	100459	100062	01-Oct-10	3	0	1	0	L	3	3	0
100445	100460	100062	01-Oct-10	3	0	0	1	R	3	0	3
100446	100461	100062	01-Oct-10	3	0	1	1	R	3	0	3
100447	100462	100062	01-Oct-10	3	0	0	2	R	3	0	3
100448	100463	100062	01-Oct-10	4	0	1	1	L	4	4	0
100449	100464	100062	01-Oct-10	3	0	1	0	L	3	3	0
100450	100465	100062	01-Oct-10	4	0	0	1	R	4	0	4
100451	100466	100063	02-Oct-10	3	0	0	2	R	3	0	3
100452	100467	100063	02-Oct-10	0	0	0	0	N	0	0	0
100453	100468	100063	02-Oct-10	3	0	0	1	R	3	0	3
100454	100469	100063	02-Oct-10	3	0	0	1	R	3	0	3
100455	100470	100063	02-Oct-10	3	0	1	1	R	3	0	3
100456	100471	100063	02-Oct-10	3	0	1	1	R	3	0	3
100457	100472	100063	02-Oct-10	3	0	0	2	R	3	0	3
100458	100473	100063	02-Oct-10	3	0	2	0	L	3	3	0
100459	100474	100063	02-Oct-10	3	0	1	1	R	3	0	3
100460	100475	100063	02-Oct-10	3	0	1	0	L	3	3	0
100461	100476	100063	02-Oct-10	3	0	1	0	L	3	3	0
100462	100477	100063	02-Oct-10	3	0	0	1	R	3	0	3
100463	100478	100063	02-Oct-10	3	0	0	2	R	3	0	3

100464	100479	100063	02-Oct-10	3	0	0	1	R	3	0	3
100465	100480	100063	02-Oct-10	3	0	0	1	R	3	0	3
100466	100481	100063	02-Oct-10	3	0	1	1	R	3	0	3
100467	100482	100063	02-Oct-10	3	0	1	1	L	3	3	0
100468	100483	100063	02-Oct-10	3	0	0	1	R	3	0	3
100469	100484	100063	02-Oct-10	3	0	2	0	L	3	3	0
100470	100485	100063	02-Oct-10	3	0	1	0	L	3	3	0
100471	100486	100063	02-Oct-10	3	0	1	1	R	3	0	3
100472	100487	100063	02-Oct-10	3	0	0	1	R	3	0	3
100473	100488	100063	02-Oct-10	3	0	0	1	R	3	0	3
100474	100489	100063	02-Oct-10	3	0	0	1	R	3	0	3
100475	100490	100063	02-Oct-10	3	0	2	0	L	3	3	0
100476	100491	100063	02-Oct-10	3	0	0	1	R	3	0	3
100477	100492	100063	02-Oct-10	2	1	2	0	L	3	3	0
100478	100493	100063	02-Oct-10	2	1	1	1	L	3	3	0
100479	100494	100064	03-Oct-10	2	2	0	2	R	4	0	4
100480	100495	100064	03-Oct-10	2	2	0	1	R	4	0	4
100481	100496	100064	03-Oct-10	2	2	0	1	R	4	0	4
100482	100497	100064	03-Oct-10	0	0	0	0	N	0	0	0
100483	100498	100064	03-Oct-10	2	2	2	0	L	4	4	0
100484	100499	100064	03-Oct-10	2	2	0	1	R	4	0	4
100485	100500	100064	03-Oct-10	0	0	0	0	N	0	0	0
100486	100501	100064	03-Oct-10	2	2	0	1	R	4	0	4
100487	100502	100064	03-Oct-10	0	0	0	0	N	0	0	0
100488	100503	100064	03-Oct-10	0	0	1	0	N	0	0	0

100489	100504	100064	03-Oct-10	2	2	1	1	L	4	4	0
100490	100505	100064	03-Oct-10	2	2	2	0	L	4	4	0
100491	100506	100064	03-Oct-10	2	2	0	1	R	4	0	4
100492	100507	100064	03-Oct-10	2	2	1	0	L	4	4	0
100493	100508	100064	03-Oct-10	2	2	0	2	R	4	0	4
100494	100509	100064	03-Oct-10	2	2	0	1	R	4	0	4
100495	100510	100064	03-Oct-10	2	2	1	0	L	4	4	0
100496	100511	100064	03-Oct-10	2	2	0	2	R	4	0	4
100497	100512	100064	03-Oct-10	4	4	1	0	L	8	8	0
100498	100513	100064	03-Oct-10	4	0	0	1	R	4	0	4
100499	100514	100064	03-Oct-10	4	0	2	0	L	4	4	0
100500	100515	100064	03-Oct-10	6	0	0	1	R	6	0	6
100501	100516	100064	03-Oct-10	0	0	0	0	N	0	0	0
100502	100517	100065	04-Oct-10	4	0	2	0	L	4	4	0
100503	100518	100065	04-Oct-10	4	0	0	1	R	4	0	4
100504	100519	100065	04-Oct-10	4	0	0	1	R	4	0	4
100505	100520	100065	04-Oct-10	4	0	2	0	L	4	4	0
100506	100521	100065	04-Oct-10	4	0	1	0	L	4	4	0
100507	100522	100065	04-Oct-10	4	0	1	0	L	4	4	0
100508	100523	100065	04-Oct-10	4	0	0	2	R	4	0	4
100509	100524	100065	04-Oct-10	4	0	0	2	R	4	0	4
100510	100525	100065	04-Oct-10	4	2	1	0	L	6	6	0
100511	100526	100065	04-Oct-10	5	2	0	2	R	7	0	7
100512	100527	100065	04-Oct-10	5	2	0	1	R	7	0	7
100513	100528	100065	04-Oct-10	5	3	1	1	L	8	8	0

100514	100529	100065	04-Oct-10	5	3	0	2	R	8	0	8
100515	100530	100065	04-Oct-10	9	0	1	0	L	9	9	0
100516	100531	100065	04-Oct-10	8	0	2	0	L	8	8	0
100517	100532	100065	04-Oct-10	8	0	0	1	R	8	0	8
100518	100533	100066	05-Oct-10	8	0	0	2	R	8	0	8
100519	100534	100066	05-Oct-10	6	0	1	1	R	6	0	6
100520	100535	100066	05-Oct-10	6	0	0	1	R	6	0	6
100521	100536	100066	05-Oct-10	7	0	2	0	L	7	7	0
100522	100537	100066	05-Oct-10	8	0	0	1	R	8	0	8
100523	100538	100066	05-Oct-10	6	0	1	0	L	6	6	0
100524	100539	100066	05-Oct-10	6	0	1	0	L	6	6	0
100525	100540	100066	05-Oct-10	6	0	1	0	L	6	6	0
100526	100541	100067	07-Oct-10	5	2	1	1	L	7	7	0
100527	100542	100067	07-Oct-10	7	0	1	0	L	7	7	0
100528	100543	100067	07-Oct-10	7	0	0	1	R	7	0	7
100529	100544	100067	07-Oct-10	5	1	1	1	L	6	6	0
100530	100545	100067	07-Oct-10	5	1	2	0	L	6	6	0
100531	100546	100067	07-Oct-10	5	1	1	0	L	6	6	0
100532	100547	100067	07-Oct-10	4	2	0	2	R	6	0	6
100533	100548	100067	07-Oct-10	4	2	1	1	L	6	6	0
100534	100549	100067	07-Oct-10	4	2	0	1	R	6	0	6
100535	100550	100067	07-Oct-10	4	2	1	1	L	6	6	0
100536	100551	100067	07-Oct-10	3	3	0	1	R	6	0	6
100537	100552	100068	08-Oct-10	6	0	0	1	R	6	0	6
100538	100553	100068	08-Oct-10	6	0	1	0	L	6	6	0

100539	100554	100068	08-Oct-10	6	0	1	1	L	6	6	0
100540	100555	100068	08-Oct-10	6	0	0	2	R	6	0	6
100541	100556	100068	08-Oct-10	6	0	0	1	R	6	0	6
100542	100557	100068	08-Oct-10	6	0	1	1	L	6	6	0
100543	100558	100068	08-Oct-10	6	0	1	1	R	6	0	6
100544	100559	100068	08-Oct-10	6	0	1	0	L	6	6	0
100545	100560	100068	08-Oct-10	6	0	1	1	R	6	0	6
100546	100561	100068	08-Oct-10	6	0	0	1	R	6	0	6
100547	100562	100069	09-Oct-10	5	0	1	0	L	5	5	0
100548	100563	100069	09-Oct-10	5	0	1	0	L	5	5	0
100549	100564	100069	09-Oct-10	5	0	0	1	R	5	0	5
100550	100565	100069	09-Oct-10	5	0	0	2	R	5	0	5
100551	100566	100069	09-Oct-10	5	0	1	0	L	5	5	0
100552	100567	100069	09-Oct-10	5	0	0	1	R	5	0	5
100553	100568	100069	09-Oct-10	5	0	0	3	R	5	0	5
100554	100569	100069	09-Oct-10	5	0	2	0	L	5	5	0
100555	100570	100069	09-Oct-10	5	0	0	1	R	5	0	5
100556	100571	100069	09-Oct-10	5	0	1	0	L	5	5	0
100557	100572	100069	09-Oct-10	5	0	3	1	L	5	5	0
100558	100573	100069	09-Oct-10	5	0	1	0	L	5	5	0
100567	100582	100071	10-Oct-10	6	0	0	1	R	6	0	6
100568	100583	100071	10-Oct-10	6	0	1	0	L	6	6	0
100569	100584	100071	10-Oct-10	6	0	0	1	R	6	0	6
100570	100585	100071	10-Oct-10	6	0	0	2	R	6	0	6
100571	100586	100072	11-Oct-10	6	0	1	1	L	6	6	0

100572	100587	100072	11-Oct-10	6	0	1	1	L	6	6	0
100573	100588	100072	11-Oct-10	6	0	2	0	L	6	6	0
100574	100589	100072	11-Oct-10	6	0	0	1	R	6	0	6
100575	100590	100072	11-Oct-10	6	0	1	1	L	6	6	0
100576	100591	100072	11-Oct-10	6	0	0	2	R	6	0	6
100577	100592	100072	11-Oct-10	6	0	1	0	R	6	0	6
100578	100593	100073	12-Oct-10	6	0	0	1	R	6	0	6
100579	100594	100073	12-Oct-10	6	0	1	0	L	6	6	0
100580	100595	100073	12-Oct-10	6	0	1	0	L	6	6	0
100581	100596	100073	12-Oct-10	6	0	2	0	L	6	6	0
100582	100597	100073	12-Oct-10	6	0	0	1	R	6	0	6
100583	100598	100073	12-Oct-10	6	0	1	1	L	6	6	0
100584	100599	100073	12-Oct-10	6	0	0	2	R	6	0	6
100620	100635	100078	17-Oct-10	6	0	1	0	L	6	6	0
100621	100636	100078	17-Oct-10	6	0	0	1	R	6	0	6
100622	100637	100078	17-Oct-10	6	0	1	1	R	6	0	6
100623	100638	100078	17-Oct-10	6	0	1	1	L	6	6	0
100624	100639	100078	17-Oct-10	6	0	0	1	R	6	0	6
100625	100640	100078	17-Oct-10	6	0	1	1	R	6	0	6
100626	100641	100078	17-Oct-10	6	0	2	0	L	6	6	0
100627	100642	100078	17-Oct-10	6	0	1	1	L	6	6	0
100628	100643	100078	17-Oct-10	6	0	2	0	L	6	6	0
100629	100644	100078	17-Oct-10	6	0	1	0	L	6	6	0
100630	100645	100078	17-Oct-10	6	0	1	0	L	6	6	0
100631	100646	100078	17-Oct-10	6	0	2	0	L	6	6	0

100632	100647	100079	18-Oct-10	6	0	1	0	R	6	0	6
100633	100648	100079	18-Oct-10	6	0	1	0	L	6	6	0
100634	100649	100079	18-Oct-10	6	0	1	1	R	6	0	6
100635	100650	100079	18-Oct-10	6	0	0	1	R	6	0	6
100636	100651	100079	18-Oct-10	6	0	1	0	RL	6	2	4
100637	100652	100079	18-Oct-10	6	0	0	2	R	6	0	6
100638	100653	100079	18-Oct-10	6	0	0	1	L	6	6	0
100639	100654	100079	18-Oct-10	6	0	0	1	L	6	6	0
100640	100655	100079	18-Oct-10	6	0	2	0	L	6	6	0
100641	100656	100079	18-Oct-10	6	0	0	1	R	6	0	6
100642	100657	100080	19-Oct-10	7	0	0	2	R	7	0	7
100643	100658	100080	19-Oct-10	7	0	0	1	R	7	0	7
100644	100659	100080	19-Oct-10	7	0	0	1	R	7	0	7
100645	100660	100080	19-Oct-10	7	0	0	1	R	7	0	7
100646	100661	100081	20-Oct-10	5	0	1	1	R	5	0	5
100647	100662	100081	20-Oct-10	5	0	0	1	R	5	0	5
100648	100663	100081	20-Oct-10	5	0	0	1	R	5	0	5
100649	100664	100081	20-Oct-10	5	0	0	1	R	5	0	5
100650	100665	100081	20-Oct-10	5	0	1	0	L	5	5	0
100651	100666	100081	20-Oct-10	0	0	0	1	N	0	0	0
100652	100667	100081	20-Oct-10	5	0	1	0	L	5	5	0
100653	100668	100081	20-Oct-10	5	0	0	2	R	5	0	5
100654	100669	100081	20-Oct-10	0	0	1	0	N	0	0	0
100655	100670	100082	21-Oct-10	5	0	1	0	L	5	5	0
100656	100671	100082	21-Oct-10	5	0	1	0	L	5	5	0

100657	100672	100082	21-Oct-10	5	0	1	1	R	5	0	5
100658	100673	100082	21-Oct-10	5	0	0	1	R	5	0	5
100659	100674	100082	21-Oct-10	5	0	0	1	R	5	0	5
100660	100675	100082	21-Oct-10	5	0	2	0	L	5	5	0
100661	100676	100082	21-Oct-10	5	0	2	0	L	5	5	0
100662	100677	100082	21-Oct-10	5	0	0	0	L	5	5	0
100663	100678	100082	21-Oct-10	5	0	0	1	R	5	0	5
100664	100679	100082	21-Oct-10	4	0	1	0	L	4	4	0
100665	100680	100082	21-Oct-10	8	0	1	0	L	8	8	0
100666	100681	100082	21-Oct-10	4	0	1	0	L	4	4	0
100667	100682	100082	21-Oct-10	3	0	0	1	R	3	0	3
100668	100683	100082	21-Oct-10	5	0	0	1	L	5	5	0
100669	100684	100082	21-Oct-10	5	0	1	1	R	5	0	5
100670	100685	100082	21-Oct-10	5	0	1	0	L	5	5	0
100671	100686	100082	21-Oct-10	5	0	0	2	R	5	0	5
100672	100687	100082	21-Oct-10	5	0	0	1	R	5	0	5
100673	100688	100082	21-Oct-10	5	0	1	0	L	5	5	0
100674	100689	100082	21-Oct-10	5	0	0	1	R	5	0	5
100675	100690	100082	21-Oct-10	5	0	0	1	R	5	0	5
100676	100691	100082	21-Oct-10	5	0	1	0	L	5	5	0

Non-Cleavage=n-Cleavage; Ovulation Point of Right=OPR; Ovulation Point of Left=OPL; N=no transfer; R=right; L=left; Right Side=RS; Left Side=LS

Table S7. Statistics on the Tβ4 overexpression (Tβ4-OE) Clonal Goat of P0

Clonal NO.	Transfer NO.	Embryo NO.	Type	Sex	Birthdate	Death date
10020	100440	100062	P（K6.1）-Tβ4	female	28-Feb-11	01-Jun-16
10030	100454	100063	P（K6.1）-Tβ4	female	17-Mar-11	Fetal Death
10027	100516	100065	P（K6.1）-Tβ4	female	11-Mar-11	
10022	100517	100065	P（K6.1）-Tβ4	female	09-Mar-11	01-Jun-15
10023	100537	100068	P（K6.1）-Tβ4	female	09-Mar-11	
10024	100537	100068	P（K6.1）-Tβ4	female	09-Mar-11	
10025	100542	100068	P（K6.1）-Tβ4	female	09-Mar-11	
10028	100550	100069	P（K6.1）-Tβ4	female	14-Mar-11	01-Jun-16
10026	100568	100071	P（K6.1）-Tβ4	female	10-Mar-11	
10041	100622	100078	P（K6.1）-Tβ4	male	24-Mar-11	15-Sep-15
10034	100633	100079	P（K6.1）-Tβ4	male	20-Mar-11	
10031	100637	100079	P（K6.1）-Tβ4	male	18-Mar-11	15-Sep-15
10029	100642	100080	P（K6.1）-Tβ4	male	17-Mar-11	
10032	100643	100080	P（K6.1）-Tβ4	male	18-Mar-11	
10036	100647	100081	P（K6.1）-Tβ4	male	21-Mar-11	
10035	100652	100081	P（K6.1）-Tβ4	male	20-Mar-11	Fetal Death
10042	100662	100082	P（K6.1）-Tβ4	male	24-Mar-11	Fetal Death
10043	100667	100082	P（K6.1）-Tβ4	male	25-Mar-11	15-Sep-15
10044	100669	100082	P（K6.1）-Tβ4	male	27-Mar-11	Fetal Death

Table S8. Statistics on the wild type (WT) Embryo of P0

Embryo NO.	Year	Cleavage
100070	2010	32
100086	2010	53
100087	2010	58
100088	2010	50
100089	2010	41
100091	2010	35
100092	2010	14
100093	2010	28
100094	2010	49

Table S9. Statistics on the wild type (WT) Oestrus of P0

Oestrus NO.	Surrogate NO.	Date
100574	100489	08-Oct-10
100575	100490	08-Oct-10
100576	100491	08-Oct-10
100577	100492	08-Oct-10
100578	100493	08-Oct-10
100579	100494	08-Oct-10
100580	100495	08-Oct-10
100581	100496	08-Oct-10
100748	100433	23-Oct-10
100749	100434	23-Oct-10
100750	100450	23-Oct-10
100751	100451	23-Oct-10
100752	100455	23-Oct-10
100753	100454	23-Oct-10
100754	100417	23-Oct-10
100755	100583	24-Oct-10
100756	100420	24-Oct-10
100757	100464	24-Oct-10
100758	100584	24-Oct-10
100759	100585	24-Oct-10
100760	100456	24-Oct-10
100761	100459	24-Oct-10
100762	100586	24-Oct-10
100763	100587	24-Oct-10
100764	100588	25-Oct-10
100765	100466	25-Oct-10
100766	100589	25-Oct-10
100767	100356	25-Oct-10
100768	100383	25-Oct-10
100769	100428	25-Oct-10
100770	100465	25-Oct-10
100771	100590	25-Oct-10
100772	100471	26-Oct-10
100773	100462	26-Oct-10
100774	100591	26-Oct-10
100775	100486	26-Oct-10
100776	100592	26-Oct-10
100777	100460	26-Oct-10
100782	100487	27-Oct-10

100783	100475	27-Oct-10
100784	100492	27-Oct-10
100785	100461	27-Oct-10
100786	100489	27-Oct-10
100787	100481	28-Oct-10
100788	100491	28-Oct-10
100789	100506	29-Oct-10
100790	100493	29-Oct-10
100791	100495	29-Oct-10
100792	100490	29-Oct-10
100793	100502	30-Oct-10
100794	100505	30-Oct-10
100795	100503	30-Oct-10
100796	100335	30-Oct-10
100797	100594	30-Oct-10
100798	100595	30-Oct-10
100799	100501	30-Oct-10

Table S10. Statistics on the wild type (WT) Transfer of P0

Transfer NO.	Oestrus NO.	Embryo NO.	Date	Embryo of Transfer		Site of Transfer			Number of Transfer		
				n-Cleavage	Cleavage	OPR	OPL	Site	Total	RS	LS
100559	100574	100070	10-Oct-10	4	0	0	1	R	4	0	4
100560	100575	100070	10-Oct-10	4	0	3	1	L	4	4	0
100561	100576	100070	10-Oct-10	4	0	0	1	R	4	0	4
100562	100577	100070	10-Oct-10	4	0	1	0	L	4	4	0
100563	100578	100070	10-Oct-10	4	0	1	0	L	4	4	0
100564	100579	100070	10-Oct-10	4	0	0	2	R	4	0	4
100565	100580	100070	10-Oct-10	4	0	0	1	R	4	0	4
100566	100581	100070	10-Oct-10	4	0	1	0	L	4	4	0
100733	100748	100086	25-Oct-10	7	0	0	1	R	7	0	7
100734	100749	100086	25-Oct-10	7	0	0	1	R	7	0	7
100735	100750	100086	25-Oct-10	7	0	0	2	R	7	0	7
100736	100751	100086	25-Oct-10	8	0	1	0	L	8	8	0
100737	100752	100086	25-Oct-10	8	0	1	0	R	8	0	8
100738	100753	100086	25-Oct-10	8	0	0	1	R	8	0	8
100739	100754	100086	25-Oct-10	8	0	0	1	R	8	0	8
100740	100755	100087	26-Oct-10	6	0	1	0	L	6	6	0
100741	100756	100087	26-Oct-10	6	0	1	1	L	6	6	0
100742	100757	100087	26-Oct-10	6	0	1	0	L	6	6	0
100743	100758	100087	26-Oct-10	6	0	0	1	R	6	0	6
100744	100759	100087	26-Oct-10	6	0	1	1	L	6	6	0
100745	100760	100087	26-Oct-10	7	0	1	1	R	7	0	7

100746	100761	100087	26-Oct-10	8	0	1	2	L	8	8	0
100747	100762	100087	26-Oct-10	7	0	1	0	L	7	7	0
100748	100763	100087	26-Oct-10	6	0	0	2	R	6	0	6
100749	100764	100088	27-Oct-10	6	0	0	1	R	6	0	6
100750	100765	100088	27-Oct-10	6	0	1	0	L	6	6	0
100751	100766	100088	27-Oct-10	6	0	1	0	L	6	6	0
100752	100767	100088	27-Oct-10	6	0	1	0	L	6	6	0
100753	100768	100088	27-Oct-10	6	0	1	0	L	6	6	0
100754	100769	100088	27-Oct-10	7	0	0	1	R	7	0	7
100755	100770	100088	27-Oct-10	6	0	0	1	R	6	0	6
100756	100771	100088	27-Oct-10	7	0	1	0	L	7	7	0
100757	100772	100089	28-Oct-10	7	0	1	0	L	7	7	0
100758	100773	100089	28-Oct-10	7	0	1	0	L	7	7	0
100759	100774	100089	28-Oct-10	7	0	0	1	R	7	0	7
100760	100775	100089	28-Oct-10	7	0	0	2	R	7	0	7
100761	100776	100089	28-Oct-10	7	0	1	1	L	7	7	0
100762	100777	100089	28-Oct-10	6	0	0	3	R	6	0	6
100767	100782	100091	29-Oct-10	7	0	1	1	R	7	0	7
100768	100783	100091	29-Oct-10	7	0	2	0	L	7	7	0
100769	100784	100091	29-Oct-10	7	0	1	1	L	7	7	0
100770	100785	100091	29-Oct-10	7	0	0	0	R	7	0	7
100771	100786	100091	29-Oct-10	7	0	1	0	L	7	7	0
100772	100787	100092	30-Oct-10	7	0	0	1	R	7	0	7
100773	100788	100092	30-Oct-10	7	0	1	0	L	7	7	0
100774	100789	100093	31-Oct-10	7	0	1	0	L	7	7	0

100775	100790	100093	31-Oct-10	7	0	2	0	R	7	0	7
100776	100791	100093	31-Oct-10	7	0	1	0	L	7	7	0
100777	100792	100093	31-Oct-10	7	0	1	0	L	7	7	0
100778	100793	100094	01-Nov-10	7	0	0	1	R	7	0	7
100779	100794	100094	01-Nov-10	7	0	0	1	R	7	0	7
100780	100795	100094	01-Nov-10	7	0	0	1	R	7	0	7
100781	100796	100094	01-Nov-10	7	0	0	1	R	7	0	7
100782	100797	100094	01-Nov-10	7	0	1	0	L	7	7	0
100783	100798	100094	01-Nov-10	7	0	0	1	R	7	0	7
100784	100799	100094	01-Nov-10	7	0	2	0	L	7	7	0

Non-Cleavage=n-Cleavage; Ovulation Point of Right=OPR; Ovulation Point of Left=OPL; N=no transfer; R=right; L=left; Right Side=RS; Left Side=LS

Table S11. Statistics on the wild type (WT) Clonal Goat of P0

Clonal NO.	Transfer NO.	Embryo NO.	Type	Sex	Birthdate	Death date
10021	100566	100070	NULL	male	08-Mar-11	01-Jun-16
10037	100744	100087	NULL	female	23-Mar-11	08-Mar-15
10038	100743	100087	NULL	female	23-Mar-11	
10045	100768	100091	NULL	male	27-Mar-11	
10046	100739	100086	NULL	male	27-Mar-11	Fetal Death
10047	100773	100092	NULL	male	27-Mar-11	Fetal Death
10048	100752	100088	NULL	male	29-Mar-11	Fetal Death
10049	100784	100094	NULL	male	01-Apr-11	15-Sep-15
